# Cement Embolus in a Patient With Multiple Myeloma: A Case Report

**DOI:** 10.7759/cureus.88940

**Published:** 2025-07-28

**Authors:** Zelinda Taylor, Sarah Lee, Priya Antil, Didar Singh

**Affiliations:** 1 Internal Medicine, Southern Illinois University School of Medicine, Springfield, USA; 2 Orthopaedics and Rehabilitation, Pandit Bhagwat Dayal Sharma Post Graduate Institute of Medical Sciences (PGIMS), Rohtak, IND; 3 Hospital Medicine, Springfield Clinic, Springfield, USA; 4 Internal Medicine, Springfield Memorial Hospital, Southern Illinois University, Springfield, USA; 5 Internal Medicine, Saint John's Hospital, Southern Illinois University, Springfield, USA

**Keywords:** anticoagulation, copd, kyphoplasty, multiple myeloma, pulmonary embolism, rsv

## Abstract

Multiple myeloma consists of characteristic symptoms such as hypercalcemia, renal insufficiency, anemia, and bone abnormalities. Kyphoplasty, a minimally invasive surgical intervention, is a commonly used procedure to stabilize bone lesions in patients with multiple myeloma. Despite its relative safety, a potential complication is cement embolism. This can occur if cement leaks into the paravertebral venous system and lodges in distant arteries. It is often asymptomatic and found incidentally on imaging studies. Occasionally, it can also present with dyspnea, chest discomfort, or acute respiratory distress syndrome. This case report highlights a 70-year-old male patient with multiple myeloma who underwent kyphoplasty and developed a pulmonary cement embolism. The treatment options for cement embolism depend on the location, size, and severity of the symptoms, with careful consideration of comorbidities and other potential causes of respiratory distress.

## Introduction

In 2021, the prevalence of multiple myeloma was 179,063 in the United States, and the lifetime average risk is 1 in 103 for men and 1 in 131 for women [1,2]. Multiple myeloma is a proliferation of plasma cells that produces monoclonal immunoglobulin [3]. Patients commonly have a constellation of symptoms, including hypercalcemia, renal failure, anemia, and bone lesions. These are often osseous lytic lesions that can lead to significant pain and potential fractures in patients [4].

Surgical intervention of these lesions with kyphoplasty and vertebroplasty offers patients improvement in pain and physical functioning [5], with studies finding an 87% and 92% reduction in pain following vertebroplasty and kyphoplasty, respectively [6]. Although these procedures can be life-changing for patients, they come with their own set of risks. Some of these risks include osteomyelitis, polymethylmethacrylate (PMMA) leakage, pulmonary cement embolism, cerebral cement embolism, and cerebrovascular collapse [5].

The patient’s unique anatomy, fracture pattern of the lesion, technique used, viscosity, and amount of the PMMA all contribute to the risk of adverse effects [7]. Of interest is the risk of pulmonary cement embolism, as these are often asymptomatic and incidental findings on future imaging studies. The prevalence of pulmonary cement embolism following kyphoplasty is between 2% and 26% [8].

A study in 2018 examined the incidence of pulmonary cement embolism in cancer patients following vertebroplasty and found that patients with multiple myeloma, compared to those with other malignancies, were more likely to develop pulmonary cement embolism [9]. This case report highlights a patient with pulmonary cement embolism following kyphoplasty, with a history of multiple myeloma.

## Case presentation

A 70-year-old man with a history of multiple myeloma, currently undergoing maintenance chemotherapy, presented to the Emergency Department with two weeks of progressive dyspnea that had significantly worsened over the past two days. His medical history was significant for hypertension, hyperlipidemia, heart failure with preserved ejection fraction (HFpEF) with an echocardiogram from two months prior showing an ejection fraction of 55%-60%, pulmonary hypertension, chronic obstructive pulmonary disease (COPD), chronic kidney disease stage III with secondary hyperparathyroidism, cirrhosis, type II diabetes, obstructive sleep apnea, atrial fibrillation on rivaroxaban, atrial flutter, gout, and arthritis. His dyspnea had worsened to the point where he felt he could not catch his breath. He also complained of a dry cough, fatigue, and mild swelling in his lower extremities bilaterally. He did not report chest pain, fever, chills, lightheadedness, syncope, headache, abdominal pain, vomiting, diarrhea, or a history of recent travel.

**Figure 1:**
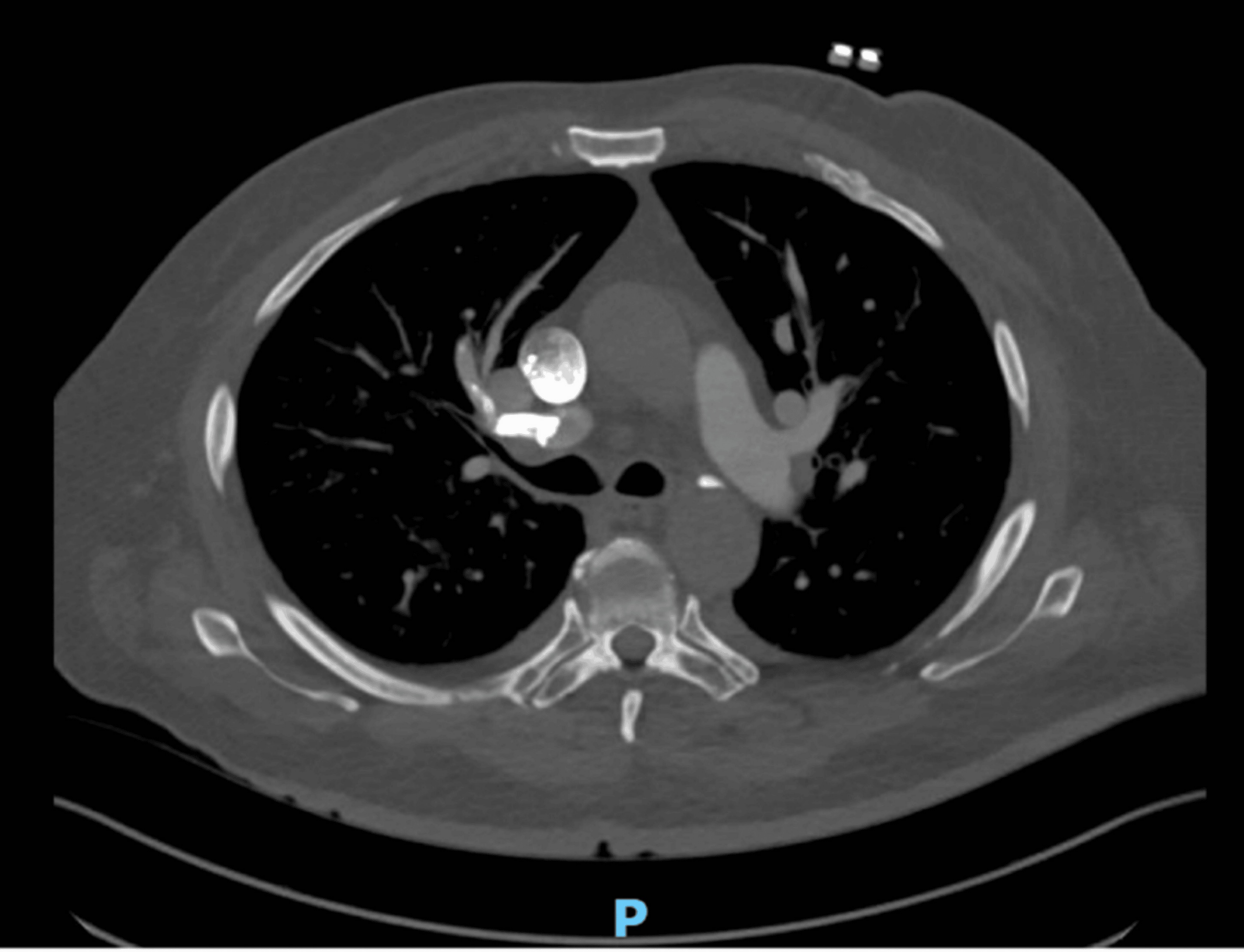
Axial CT demonstrating a hyperdense cement embolism in the right upper lobe pulmonary artery CT, computed tomography

In the emergency room, a complete blood count, complete metabolic panel, troponin, brain natriuretic peptide, procalcitonin, COVID polymerase chain reaction (PCR), influenza PCR, respiratory syncytial virus (RSV) PCR, lactic acid, chest X-ray, computed tomography angiography (CTA) chest, and electrocardiogram (EKG) were ordered. The patient’s oxygen saturation on admission was 84% on room air and improved to 100% with 2 L of oxygen. He was tachypneic and in moderate respiratory distress, with decreased breath sounds and diffuse expiratory wheezing. RSV PCR was positive. CTA showed new hyperdense material in the lumen of the right upper lobe pulmonary artery, extending into its subsegmental branches in the anterior and lateral right upper lobe and into a few subsegmental pulmonary arteries in the right middle lobe, suggestive of pulmonary cement embolization (Figure 1). Significant lab results are included in Table 1. All other reports came back unremarkable, and the patient was admitted to the general hospital floor.

**Table 1:** Patient's initial hospitalization laboratory results BNP, brain-type natriuretic peptide; WBC, white blood cell

Lab reports	Results	Reference range
WBC	5.6	3.4-9.4
Troponin (pg/mL)	41.9	0-20
BNP (pg/mL)	701	0-63
Lactic acid (mmol/L)	3.2	0.5-2.0
HCO_3_ (mmol/L)	30.3	24-28
Procalcitonin (ng/mL)	0.33	0-0.49

On his first day of hospitalization, a discussion with the patient and his wife confirmed that, one month prior, the patient had undergone radiofrequency ablation and balloon-assisted kyphoplasty of the T10 and T12 vertebrae with Interventional Radiology. He recovered well from the procedure without any complications. Two weeks after the procedure, he began to experience some dyspnea. Per the interventional radiologist, the patient had already been on rivaroxaban 15 mg daily for atrial fibrillation, and the dose had been held for three days prior to the procedure and then resumed afterward. It was determined that the cement embolism would not be treatable with anything less than a very invasive surgery, and he would likely benefit from therapeutic anticoagulation. The patient’s oncologist was also notified, and a collective decision was made to increase the rivaroxaban from 15 mg daily to 15 mg twice daily for three weeks to mitigate partial vessel occlusion and reduce the risk of potential thrombosis. Following the three weeks of the increased dose of rivaroxaban 15 mg twice daily, the patient's home dose was then increased to 20 mg daily. 

The rest of the patient’s three-day hospital stay was relatively uneventful. He continued to require supplemental oxygen and was discharged home on 2 L of oxygen via nasal cannula. Unfortunately, the patient returned to the Emergency Department three times in the following six weeks - for a fall, weakness and diarrhea, and shortness of breath - and was hospitalized for the latter two Emergency Department encounters. Four months after the hospital stay described in this case study, his multiple myeloma was found to have relapsed, and the patient passed away two months later.

## Discussion

Current guidelines from the Society of Interventional Radiology state that anticoagulation recommendations following kyphoplasty and vertebroplasty are patient-specific [10]. Anticoagulation is recommended in patients with atrial fibrillation, mechanical heart valves, and a history of venous thromboembolic events [10]. For patients without these conditions, anticoagulation decisions are based on patient age, body mass index, postprocedural risk of bleeding, and renal function [10]. Spinal procedures are categorized as high bleeding risk; therefore, patients’ platelet counts and international normalized ratio (INR) are routinely checked prior to surgery [10]. A fine balance must be taken into consideration regarding anticoagulation following these high bleeding risk procedures. 

In a study published in *Cancer Imaging*, 102 patients with a pathology-confirmed cancer diagnosis were followed after vertebroplasty, and of these patients, 37 (36.3%) had multiple myeloma [9]. It was found that 13 of the 102 patients had a cement pulmonary embolism following kyphoplasty, with 11 of those cases diagnosed via CT scan [9]. Although cement pulmonary embolism was found on imaging, 61.5% of patients were asymptomatic [9]. The study found that cement pulmonary embolism was more common in patients with multiple myeloma compared to those with other malignancies [9]. While all multiple myeloma patients who developed cement pulmonary embolism underwent vertebroplasty due to malignant tumor infiltration, others had vertebroplasty due to osteoporosis [9]. The study found that death in these patients was not associated with cement pulmonary embolism [9]. 

Since the procedure is conducted using fluoroscopy, imaging studies such as CT scans and chest X-rays are usually not performed following the procedure. In the case of cement extravasation, these studies may be useful in immediately identifying complications and treating them before they become symptomatic. As stated in the study in *Cancer Imaging*, a majority of patients with cement pulmonary embolism are asymptomatic [9]. In patients who have an increased risk of venous thrombosis, a CT scan of the chest would be useful following the procedure to ensure a pulmonary embolism has not formed. In patients who do develop a pulmonary embolism from the procedure, there is a risk that it contains PMMA [5]. For these patients, complete resolution of the embolism would only occur following surgical removal. In our patient, surgery would have been required to remove the cement embolus due to its location. This surgery would have been very invasive and was not recommended, especially due to the patient’s multiple comorbidities. At that time, he was diagnosed with RSV bronchiolitis, and the cement emboli finding on CT was most likely an incidental finding - not the primary cause of his acute dyspnea. The patient also had comorbid COPD and likely had dyspnea at baseline. This contributed to difficulty in deciphering whether his dyspnea was due to his underlying medical conditions or something new and more concerning, such as a pulmonary embolism.

The patient was already on rivaroxaban for atrial fibrillation anticoagulation, but he was at an increased risk for hypercoagulability due to his multiple myeloma and current treatment with lenalidomide [11]. The dosage for anticoagulation in the setting of atrial fibrillation is less than the venous thromboembolism therapeutic dosage [12]. There is currently no definite protocol regarding anticoagulation in patients with cancer who are actively undergoing chemotherapy. For this patient, it was recommended to increase his rivaroxaban dosage to the venous thromboembolism therapeutic dose to prevent him from developing worsening symptoms and progressive pulmonary embolism. The patient's glomerular filtration rate (GFR) on admission was 59, so appropriate renal dosing was considered. 

In patients without underlying hypercoagulability, imaging could be completed following the kyphoplasty to help identify patients who are at a higher risk of developing complications. Patients with concern for thromboembolic events may be started on a prophylactic direct oral anticoagulant, such as dabigatran, after kyphoplasty and vertebroplasty, but our patient would have needed lower renal dosing and his partial thromboplastin time monitored due to his chronic kidney disease [10]. Another option for prophylactic anticoagulation would be warfarin, but this would require a larger time commitment from the patient in terms of repeated laboratory studies and dietary restrictions. 

## Conclusions

Although kyphoplasty and vertebroplasty are helpful procedures to reduce spinal pain in patients with multiple myeloma, there are significant risks that need to be taken into consideration. This patient had multiple comorbidities, which further contribute to the potential risks and difficulty in identifying new-onset complications. This case report highlights the difficulties of identifying the cause of new-onset dyspnea post-kyphoplasty in an individual with multiple comorbidities. While the exact mechanism of the patient’s dyspnea - between the pulmonary cement embolism and the RSV bronchiolitis - is unknown, it is possible that each is playing a role. If the patient were to have a future kyphoplasty, it would be recommended to start therapeutic anticoagulation or add an antiplatelet agent after the procedure to prevent an additional pulmonary cement embolism. Overall, this report contributes to the discussion on postoperative care protocols for kyphoplasty, especially in individuals with multiple myeloma and multiple comorbidities.
